# Editorial: Organohalide respiration: New findings in metabolic mechanisms and bioremediation applications, Volume II

**DOI:** 10.3389/fmicb.2022.1112309

**Published:** 2022-12-15

**Authors:** Shanquan Wang, Jianzhong He, Chaofeng Shen, Michael J. Manefield

**Affiliations:** ^1^Environmental Microbiome Research Center, School of Environmental Science and Engineering, Guangdong Provincial Key Laboratory of Environmental Pollution Control and Remediation Technology, Southern Marine Science and Engineering Guangdong Laboratory (Zhuhai), Sun Yat-sen University, Guangzhou, China; ^2^Department of Civil and Environmental Engineering, National University of Singapore, Singapore, Singapore; ^3^Department of Environmental Engineering, College of Environmental and Resource Sciences, Zhejiang University, Hangzhou, China; ^4^School of Civil and Environmental Engineering, University of New South Wales, Sydney, NSW, Australia

**Keywords:** organohalide respiration, *Dehalococcoides*, electron transport chain, reductive dehalogenase, microbiome, bioremediation

The massive production and use of organohalides resulted in their worldwide contamination in soil, sediment and other environmental matrices (He et al., 2021). Organohalide-respiring bacteria (OHRB)-mediated reductive dehalogenation not only represents a promising solution for remediation of sites contaminated by organohalides (Jugder et al., 2016; Atashgahi et al., 2018), but involves element cycling in both terrestrial and marine environments (Horna-Gray et al., 2022; Xu et al., 2022). Recent research progress in characterizing major OHRB and crystal structures of key functional enzymes provided critical insights into organohalide respiration of OHRB (Bommer et al., 2014; Payne et al., 2015; Kublik et al., 2016; Wang et al., 2018; Picott et al., 2022). Nonetheless, there are still many puzzles to be resolved for better mechanistic understanding and bioremediation applications. Therefore, this Research Topic was formulated in two volumes to solicit manuscripts related to organohalide-respiring bacteria, reductive dehalogenase (RDase) and associated electron transport chain, dehalogenating microbiome, and organohalide bioremediation. Given the success of Volume I of this Research Topic and the rapidly evolving subject area, Volume II was launched for the publication of new research findings and updated information. We selected four manuscripts for publication after a rigorous peer review process.

## Organohalide-respiring bacteria

In a *Dehalococcoides*-containing enrichment culture, Zhao et al. reported extensive and even complete debromination of two commonly used polybrominated diphenyl ethers (PBDEs, i.e., BDE47 and BDE183). In addition, the debromination extent and rate of BDE183 could be enhanced by amendment of the BDE47. This study provides knowledge on new capabilities of *Dehalococcoides* and its potential in bioremediation of sites contaminated by both DBE47 and BDE183.

## RDases and associated electron transport chains

Reductive dehalogenase is the key enzyme to catalyze halogen removal from organohalides. Based on both transcription and translation analyses, Cimmino et al. deciphered the stoichiometry of *pceABCT* individual gene products in OHRB of Firmicutes. Notably, in contrast to a previously proposed model, results showed the formation of a membrane-bound PceA_2_B that could be devoid of PceC. These results provide unprecedented insight into the electron-accepting complex in PCE-dechlorinating OHRB of the phylum of Firmicutes.

## Organohalide bioremediation

Bioelectrochemical systems (BES) hold great potential for bioremediation of sites co-contaminated by organohalides and heavy metals. Matturro et al. employed both 16S rRNA gene amplicon sequencing and metagenomic analyses to elucidate the microbial interactions among *Dehalococcoides, Methanobrevibacter* and *Methanobacterium* for the efficient dechlorination of trichloroethene (TCE) and reduction of Cr(VI) in a BES. In addition, at sites contaminated with chlorinated ethenes, abiotic factors (e.g., iron sulfide minerals) could determine the fate of chloroethenes by affecting organohalide respiration of OHRB. Li et al. reported that FeS enhanced *Dehalococcoides*-mediated reductive dechlorination of TCE by formation of FeS nanoparticles and up-regulation of *tceA* transcription. These results could guide efficient bioremediation of sites contaminated by chlorinated ethenes and other contaminants.

With the success of the two volumes of this Research Topic, we would like to thank all the authors and reviewers for their valuable contributions. These papers significantly improve our understanding in organohalide-respiring bacteria and their electron transport chains, as well as in dehalogenating microbiome and bioremediation implications. Notably, several research gaps were also highlighted in this Research Topic, and awaited future studies: (1) contribution of microbial reductive dehalogenation to attenuation of organohalides in natural environments; (2) cycling of organohalides in varied environmental matrices and associated functional microorganisms and enzymes; (3) reciprocal interactions of the commonly co-existing abiotic processes with the OHRB-mediated reductive dehalogenation process. We hope that this collection of reviews and original research articles will be helpful for researchers and engineers seeking information on organohalide respiration and bioremediation applications.

## Author contributions

All authors listed have made a substantial, direct, and intellectual contribution to the work and approved it for publication.
